# Magnetorheological Effect of Magnetoactive Elastomer with a Permalloy Filler

**DOI:** 10.3390/polym12102371

**Published:** 2020-10-15

**Authors:** Dmitry Borin, Gennady Stepanov, Anton Musikhin, Andrey Zubarev, Anton Bakhtiiarov, Pavel Storozhenko

**Affiliations:** 1 Chair of Magnetofluiddynamics, Measuring and Automation Technology, TU Dresden, 01069 Dresden, Germany; 2 State Scientific Research Institute for Chemical Technologies of Organoelement Compounds, Shosse Entuziastov 38, 111123 Moscow, Russia; gstepanov@mail.ru (G.S.); abakhtia@gmail.com (A.B.); bigpastor@mail.ru (P.S.); 3 Department of Theoretical and Mathematical Physics, Ural Federal University, Lenina Ave 51, 620083 Ekaterinburg, Russia; antoniusmagna@yandex.ru (A.M.); A.J.Zubarev@urfu.ru (A.Z.); 4 M.N. Mikheev Institute of Metal Physics of the Ural Branch of the Russian Academy of Sciences, 620108 Ekaterinburg, Russia

**Keywords:** magnetorheology, magnetoactive elastomer, ferrogel, permalloy filler, damping effect

## Abstract

Within the frames of this study, the synthesis of a permalloy to be used as a filler for magnetoactive and magnetorheological elastomers (MAEs and MREs) was carried out. By means of the mechanochemical method, an alloy with the composition 75 wt.% of Fe and 25 wt.% of Ni was obtained. The powder of the product was utilized in the synthesis of MAEs. Study of the magnetorheological (MR) properties of the elastomer showed that in a ~400 mT magnetic field the shear modulus of the MAE increased by a factor of ~200, exhibiting an absolute value of ~8 MPa. Furthermore, we obtained experimentally a relative high loss factor for the studied composite; this relates to the size and morphology of the synthesized powder. The composite with such properties is a very perspective material for magnetocontrollable damping devices. Under the action of an external magnetic field, chain-like structures are formed inside the elastomeric matrix, which is the main determining factor for obtaining a high MR effect. The effect of chain-like structures formation is most pronounced in the region of small strains, since structures are partially destroyed at large strains. A proposed theoretical model based on chain formation sufficiently well describes the experimentally observed MR effect. The peculiarity of the model is that chains of aggregates of particles, instead of individual particles, are considered.

## 1. Introduction

Magnetic gels and elastomers feature composite materials consisting of a polymer matrix and fine magnetic particles distributed inside it. Belonging to a family of multifunctional field-sensitive materials, these systems attract significant interest due to an extensive set of unique physical properties they exhibit, which are valuable for many progressive technologies and make it possible to rank them among ‘smart’ materials.

The dependence of their macroscopic rheological properties on external magnetic fields is one of their fascinating features, which is attractive from the viewpoint of practical applications and challenging for scientific study. Opening the possibility to control their behavior with the help of moderate magnetic fields ranging from ~50 to ~600 mT, it makes these composites usable as magnetocontrollable active and passive devices such as actuators and dampers [1,2,3,4,5,6,7,8,9,10,11,12,13,14,15,16,17]. Thus, employing magnetorheological/magnetoactive elastomers (MREs/MAEs) as the operating element in shock-absorbing devices seems to be a sound design concept. The pioneer publications made in 1983 dedicated to the anomalous behavior exhibited by rubbers containing magnetic filler [18] were followed by scientific reports made in 1995 by Toyota Corporation about the magnetic field-controlled variation of elasticity demonstrated by composites filled with an iron powder [19] and a number of patents and papers issued by research teams at Ford and Lord Corporations [20,21,22].

The macroscopic properties of MAE and their dependence on external magnetic field are determined by the disposition of particles in the host polymer, structures formed by them, structural transformations occurring under the influence of the field, and large-scale deformations suffered by the material. The size of the particles together with their shapes and concentration, the overall formula of the composite including the nature of the matrix polymer predetermining the stiffness of the filled elastomer [23,24,25,26,27,28,29,30,31,32,33,34,35,36,37,38], and the external magnetic and mechanical influence vectors [32] are also important factors lying behind the specifics of the field-sensitivity.

As may be noticed from the literature, among other materials, which might be used for the fabrication of magnetorheological (MR) materials including carrageenan [9], polyurethane [17,36], and copolymer gels SEBC [34], leadership has stably been held by silicone rubber [1,3,12,14,16,18,23,32,36]. Partly it is caused by the convenience of controlling the polymerization degree and thus the stiffness of the matrix [32]. The employment of spherical micro-sized carbonyl iron powders (CIPs), prepared by chemical decomposition of purified iron pentacarbonyl, is dictated by the good magnetic features demonstrated by this metal powder. Field-induced formation of CIP particle structures inside the matrix promote an increased MR effect [1,23,24,25,26,27,31,32,38].

Studies done in this area have unveiled certain interesting issues. Dedicating their investigations to the rheological characteristics of elastomers with magnetic fillers, the researchers used silicone semi-products from different sources. Yet, they might have used additional reagents to either modify the filler particles or stimulate polymerization. As a result, producing specimens of composite material with similar formulae, they predetermined the degree of attachment of the polymer to the surfaces of filler particles, which were reflected on the final damping characteristics. In view of this problem, it does not seem possible to derive a general rule unmistakably predicting the precise result upon addition of a component or changing its concentration.

Multiple experiments performed on MRE have resulted in a significant collection of research data pointing to a strong relationship between the rheological characteristics of the material and magnetic field. Partly, the results were obtained from studies on damping devices [1,2,3,4,5,6,7,8,9,10,11,12,13,14,15,16,17] or from investigations dedicated to the magnetorheological effect [23,24,25,26,27,28,29,30,31,32,33,34,35,36,37,38,39,40,41,42,43,44,45,46,47,48,49,50,51]. For instance, influenced by a field of 0.8 T, a silicone and carbonyl iron-based sample initially polymerized in a field of 1.5 T demonstrated an increase of its shear modulus (G′) from 0.25 to 2.5 MPa [1]. The authors of [2] reported that at 0.7 T, the relative and absolute MR effects exhibited by an as-prepared MRE sample were found to be 725% and 2.5 MPa, respectively, and the loss-factor was 0.32. According to [3], the application of small (0.01%) strains to a sample containing 70 wt.% of carbonyl iron caused an increase of G′ from 30 kPa at zero-field to 150 kPa at 440 mT. In a similar way, a sample subjected to compression showed an increase of its elasticity modulus by 140 kPa in a field of 125 mT; at the same time, the natural frequency of an MRE absorber working in squeeze mode can be tuned from 37 to 67 Hz by turning on a field of 312 mT [4]. The authors of [5] reported an increase of G′ from 40 to 250 kPa at 750 mT. The object of study in [8] was an MRE-based damper subjected to a mechanical influence in a magnetic field. Whereas compression and shear resulted in a 27 and 22% change of the elasticity, respectively, static tests yielded values of up to 90%. Similarly, an MR gel absorber with a tunable frequency range from 56 to 67 Hz was reported in [9]; influenced by a magnetic field of 100 mT, it exhibited a 44% change of gel stiffness. Likewise, stiffness increase observed in MRE may reach 321.5%; the resonant frequency of a semi-active/passive integrated isolator can be tuned from 30 Hz (no applied magnetic field) to 51 Hz (a magnetic field is applied) [12]. A similar effect was described in [13]: Application of a 220 mT magnetic field resulted in a shift of the resonant frequency of an MRE-based absorber from 30 to 50 Hz. Meanwhile, the authors of [14] reported an increment of 103% of natural frequency on the application of an external field. The quantitative characteristics of MRE subjected to various degrees of strain in a field of 1 T as the result of recent investigations are also interesting for comparison. Within the frames of the part of research preceding testing in the damper, the material exhibited elasticity modulus and relative magnetorheological effect values of 3–5 MPa and 58%; 1–3 MPa and 300%; and 1–2.5 MPa and 409% at a 0.01, 0.1, and 1% strain, respectively [16]. Finally, a study of a polyurethane-based composite was conducted in [17]. According to obtained results, the application of a 200 mT magnetic field caused an increase of the elasticity modulus from 0.2 to 0.6 MPa, whereas the loss-factor remained at 0.2.

At the same time, the strength of the MR effect demonstrated by MRE has been in the focus of attention of researchers for a while. For example, an isotropic carbonyl iron-filled (28 vol.%) silicone-based composite material specimen subjected to a change of magnetic field from 0 to 500 mT exhibited a relative change of the storage modulus G′ of ~2300% from the initial value 0.12 MPa [23].

Other observations indicated that samples prepared under the influence of a structuring magnetic field during polymerization exhibited an increase of the elasticity modulus and loss-factor from 4 to 8 MPa and from 0.12 to 0.22, respectively, at small (0.01%) strains and in a field of 52 mT, whereas isotropic specimens demonstrated little effect [24].

The relative MR effect (ΔG′/G′_0_) becomes more pronounced with the size of particles of the filler. For example, an isotropic sample filled with carbonyl iron particles of an average size of 3.5, 13, and 23 µm demonstrates an effect of 34, 253, and 900%, respectively, whereas that of an anistropic, i.e., field-structured during the manufacturing, specimen will be 125, 945, and 1125%, respectively [25,27].

Anisotropic composites fabricated by the authors of [21] contained 10–30 vol.% of carbonyl iron. Subjected to an increase of magnetic field from 0 to 800 mT at a strain amplitude of 1% and a frequency of 2 Hz, they exhibited ΔG′/G′_0_ values in the range of 30–39%. At the same time, the value of the loss factor decreases by 50% as the frequency changes from 2 to 20 Hz and the strain amplitude increases from 1 to 10%. It is interesting to note that the authors of [24] reported a close result (~38%) for the relative change of the loss-factor measured in similar conditions for a similar sample.

Attention to the stiffness of the polymer matrix is drawn in [29] and [31]. Prepared on the basis of ‘soft’, ‘moderate rigidity’, and ‘rigid’ polymers, the samples (isotropic, 20 vol.% of Fe) were subjected to the influence of magnetic fields in the range of 0–650 mT for the measurement of the storage and loss moduli, whose values appeared to be 4800, 123, and 46% for ΔG′/G′_0_ and 1400, 77, and 37% for ΔG″/G″_0_, which, as may be noticed, drop significantly when the matrix loses flexibility.

Within the frames of research work [32], the authors investigated the stiffening phenomenon in samples filled with magnetite (particle size 8.4 µm) as a function of interior orientation, frequency, and direction of magnetic field and load. The relative effect amounted to 50%. At the same time, even on the basis of iron fillers, it is still possible to deliver novel ideas. An example is a method of modification of the surface of spherical iron particles (size 6.5 µm) with nanoparticles of iron reduced from an aqueous solution of FeSO_4_ by NaBH_4_ given in [36]. With polyurethane selected as the matrix polymer, the highest values of ΔG′/G′_0_ ~670% and relative loss factor of −50% at a 1 T magnetic field had the sample containing 64, 6, and 30 wt.% of CIP, iron nanoparticles, and polyurethane, respectively.

An absolute and relative MR effect of 2 MPa and 284%, respectively, exhibited by the material at 290 mT, are described in [37]. At the same time, the authors of [38] also investigated the influence of the interior orientation of the composite on its performance. Whereas the maximal absolute effect shown by an isotropic and anisotropic sample in a field of 0.8 T was 0.3 and 0.8 MPa, respectively, the relative MR effect amounted to 646 and 343%, respectively. Measured at 0.8 T, the loss-factors were found to be 0.15 for a sample with no orientation and 0.20 for an oriented specimen; however, strengthening of the field caused increasing of this parameter. Relative 60% variations in the case of rigid elastomer and more than 470% variations in the case of soft elastomer were observed on increasing the external field from 0 to 0.5 T [39]. In addition, the authors of [40] found out that composites containing particles with diameters of 20 and 100 µm exhibited relative MR effects of 30 and 60% in a magnetic field of 225 mT.

In a series of publications, quite significant values of magnetorheological effect have been reported. For example, an increase of the Young modulus by a factor of 40 resulting from stretching the sample in magnetic field was observed in [41]. A raise in elasticity by almost two orders of magnitude was obtained on the rheometer in the small-constant shear deformation regime [42]. Also, samples of magnetoactive elastomer filled with big particles have been noticed to increase their Young’s modulus by 100 times in a field of 290 Oe at strains of 1–2%. The dependence of the Young modulus on deformation exhibits features of extremal functions. At small (below 0.5%) and strong (exceeding 2%) strains, the elasticity modulus falls dramatically [43]. In multiple investigations conducted by numerous scientific groups, there have been observed relative modulus variations ranging from 18 to 10,000%. At the same time, even higher relative changes of elasticity are possible in gels with moduli below 1 kPa [44]. However, this effect is rather related to a transitional condition of the liquid-gel system.

Speaking of additional examples, it is worth mentioning some values reported for a soft sample (G′_0_ = 30 kPa) containing 30 vol.% of CIP by Böse [45]: influenced by a magnetic field of 700 mT, the material showed ΔG′/G′_0_ and ΔG′ equal to ~90 and ~2.5 MPa, respectively. Measuring these parameters at 300 mT, the authors of [46] came up with 10 and 1 MPa, which is quite close. Simultaneous application of a field of 700 mT and a 1% strain resulted in a similar observation with ΔG′/G′_0_ = 90 and ΔG′ = 2.5 MPa [47,48]. Variation of polymer matrices makes it possible to obtain various outputs for ΔG′/G′_0_ and ΔG′. As has been reported in [49], samples based on polymers with different elasticities but filled with a mixture of magnetite, particle size 0.2 µm, and an iron powder, particle size 40 µm, yielded ΔG′/G′_0_ being 50 or 180 and ΔG′ equal to 5.5 or 1.5 MPa. At the same time, variation of filler composition results in certain changes. Impregnation of silicone elastomer (G′_0_ = 33 kPa) with a mixed powder containing 36 wt.% of CIP and 64 wt.% of iron particles being 60 µm in diameter and with pure CIP gave ΔG′/G′_0_ and ΔG′ values of 139 and 4.7 MPa, respectively, for the former and similarly 65 and 2.8 MPa for the latter [50]. In addition, it has been noticed that under the influence of a field of 650 mT, CIP-based elastomers exhibit ΔG′/G′_0_ and ΔG′ values of 62–390 and 4.5 MPa, respectively, depending on the polymer selected as the matrix [51].

Analysis of the literature indicates that despite the fact that investigations dedicated to the MR-effect are numerous and the overall data collection is huge, the information available does not suffice for making the complete picture. One of the reasons is that authors frequently prefer to mention the highest magnitudes only, referring to the absolute or relative effect. However, in our opinion, a quality MRE sample is expected to demonstrate both at a decent level simultaneously.

The present work is focused on the search for new compositions and study of the relationship between the formula and MR effects. In addition to the experimental study of a permalloy-based composite, we also present a theoretical model that sufficiently well predicts the MR-effect.

## 2. Methods of Production of Permalloy Fillers and Fabrication of MAE Specimens 

### 2.1. Methods Production of Permalloy Filler 

Synthesis of a magnetic filler for MAE is carried out by the mechanochemical method in a planetary mill. The synthesis was conducted in the Pulverisette 5 planetary mill (Fritsch, Weimar, Germany). A mixture of powders of carbonyl nickel (grade ПНК-УТ2, Norilsk Nickel, Moscow, Russian Federation) and carbonyl iron (grade P-10, Sintez CIP, Dzerzhinsk, Russian Federation) was loaded into the mill together with 7-mm iron beads and an organic solvent and then subjected to severe mechanochemical treatment at a rate of 350 rpm for 30 min. As a result, temperature inside the containers increased to 90 °C during the first 10 min. However, owing to cooling performed by spraying with water and air mixture, it did not grow farther. During the treatment, aggregation of Fe and Ni particles took place, accompanied by their plasticization and grinding. A series of such treatments resulted in the formation of particles mechanically melted together. The product was recovered by vacuum filtration followed by 24-h drying at 50 °C.

In order to suppress aggregation in the powders, supply them with lyophobic properties, and increase their compatibility with silicone, they are modified by treatment with a hexane solution of a mixture of the water-repelling agent (oligo-(methyl (or ethyl) hydrogen)siloxanes [RSiHO]_n_ (R = CH_3_, C_2_H_5_; *n* = 10–15)) and polydimethylsiloxane. For permalloy powders with particle sizes in the range 3–40 µm, the concentration of the modifying agent amounts to 1% of the mass of the powder. The mixture of the powders with the modifying solution is then treated on a roller dispenser and finally dried.

### 2.2. Properties of the Synthesized Permalloy Filler

**Figure 1:**
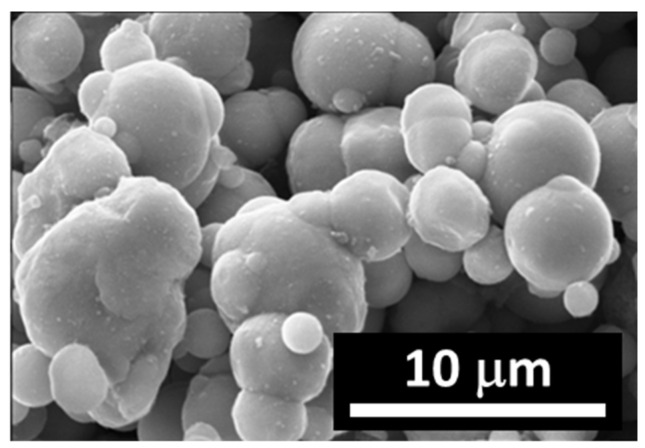
Carbonyl iron microparticles reduced in hydrogen.

**Figure 2:**
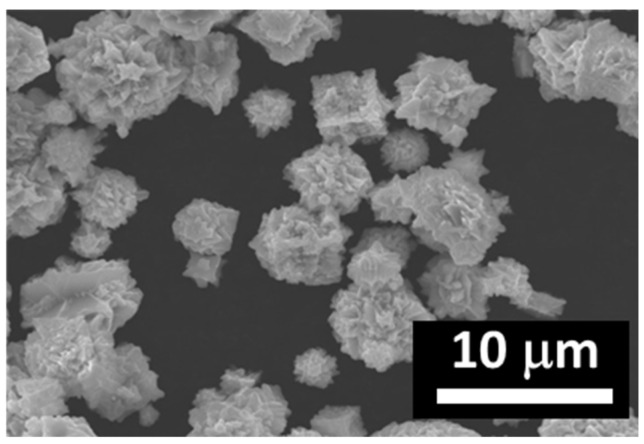
Carbonyl nickel microparticles.

**Figure 3:**
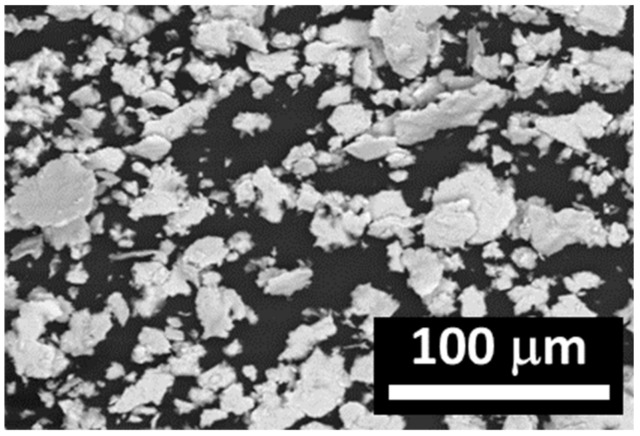
Fe/Ni alloy microparticles.

In the process of synthesis of permalloys, carbonyl iron previously subjected to reduction in hydrogen at 450 °C (Figure 1) was used. The particles are spherical with smooth surfaces. The powder of carbonyl nickel had an average particle size of 5 µm. The powder of carbonyl nickel had an average particle size of 9 µm (Figure 2). Carbonyl nickel particles possess rather irregular shapes and “furry” surfaces. Simultaneous grinding of soft iron powders with them results in the formation of Fe–Ni aggregates (Figure 3).

The obtained powders exhibited quite an extensive size range demonstrating an increase of the mean diameter to 13 µm. The permalloy particles became larger, while their surface became irregularly shaped.

**Figure 4:**
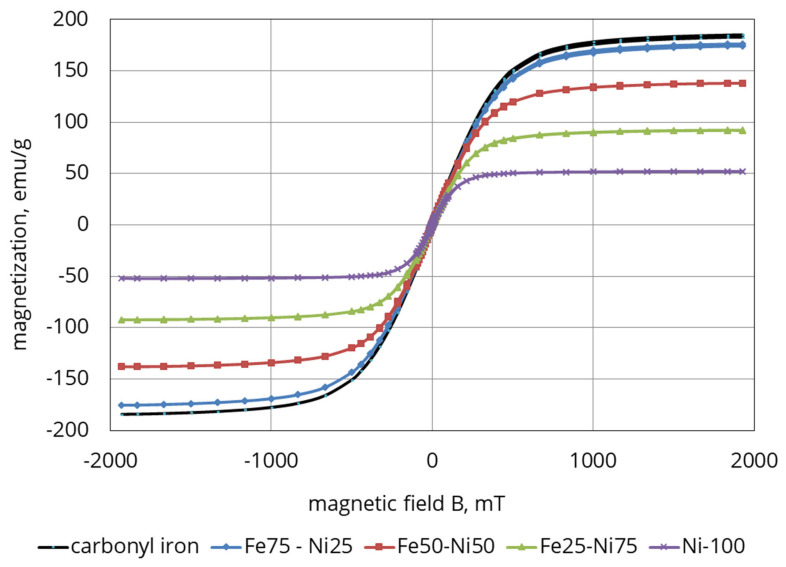
Magnetization curves of Fe–Ni systems with xFe = 0, 25, 50, 75, and 100 wt.%.

Magnetic and dispersing properties of the synthesized samples of iron-nickel alloys were investigated. The magnetic measurements were carried out using a Lake Shore 7407 vibrating sample magnetometer (Lake Shore, Westerville, OH, USA). The magnetization curves (hysteresis loops) of permalloys prepared with 25, 50, and 75 wt.% of Fe are presented together with those of pure iron and nickel in Figure 4.

As may be seen from the graph, the magnetization of the samples grows as the strength of the magnetic field increases. In the case of the 75% Fe sample, the curve practically coincides with that exhibited by carbonyl iron. It is evidently related to the treatment of the iron powder with hydrogen at 450 °C done previously, which not only removed carbides from the structure, but also improved to a certain degree its magnetization. A magnetic alloy (75% Fe/25% Ni) was chosen to prepare MAE as it has the highest magnetic properties comparing to other Fe–Ni samples. 

**Figure 5:**
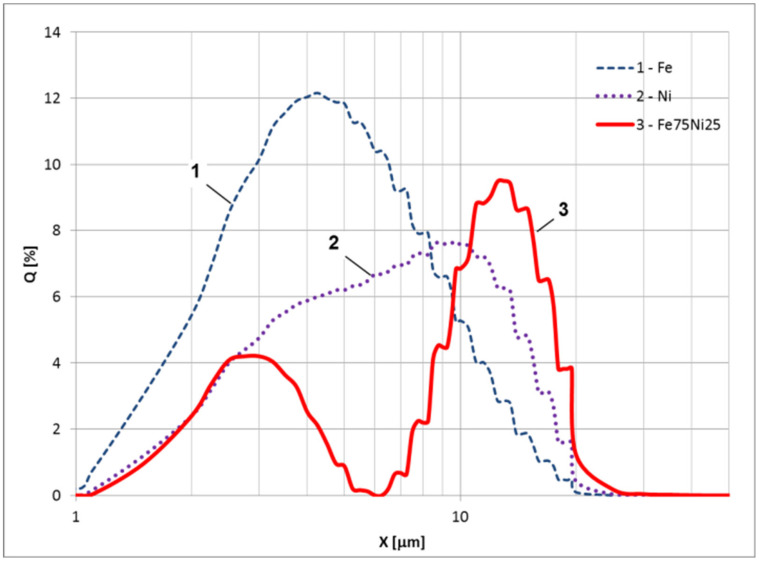
The size distribution for 1—Fe, 2—Ni and 3—Fe/Ni alloy.

The sizes of alloy (Fe/Ni), iron, and nickel powders were measured using the static light scattering with the Analysette 22 Micro Tec instrument (Fritsch, Weimar, Germany). The obtained size distribution is shown in Figure 5. The average size accordingly is: Fe—5.1 µm, Ni—9 µm, and Fe/Ni alloy—13 µm.

From the data obtained it follows that the average size of the magnetic filler (permalloy) has increased compared to the original iron and nickel. Moreover, the particles of the filler have a binary size distribution and an ellipsoidal shape. The saturation magnetization is approximately 10% lower than the magnetization of carbonyl iron. The presence of the first peak in the particle size distribution (Figure 5) may indicate that not all particles have reacted and that in addition to the Fe/Ni alloy, the powder may still also contain some proportion of carbonyl iron. A detailed chemical analysis of the powder obtained is beyond the scope of this study, but it is clear that the used mechanical synthesis process of the permalloy could be further optimized. 

### 2.3. Fabrication of Magnetoactive Elastomer

MAE samples were synthesized by mixing the two components of SIEL-resin, a product of Russian State Scientific Research Institute for Chemical Technologies of Organoelement Compounds with permalloy powder. The dispersion of the powder in the liquid polymer semi-product (oligomer) was carried out on a grinder, in which the mixture was ground between the rolls spinning towards each other at different rates. The liquid composition that was obtained was poured into molds and sent for polymerization. After preparation of the mixture and heating it above 100 °C, the process of polymerization begun. It is described in detail in [52]. The concentration of the filler was 30 vol.%. The MAE samples had cylindrical shapes with 15 and 2 mm in diameter and height, respectively.

## 3. Magnetorheological Properties of MAE

**Figure 6:**
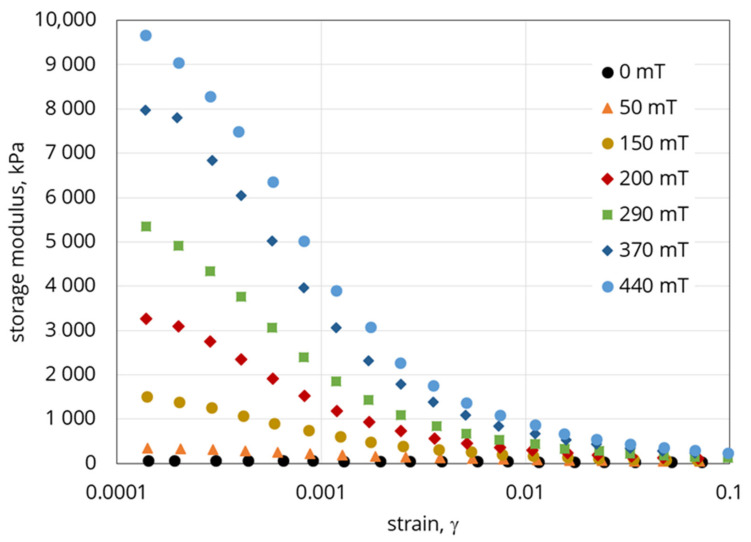
Shear modulus as a function of deformation in various magnetic fields.

**Figure 7:**
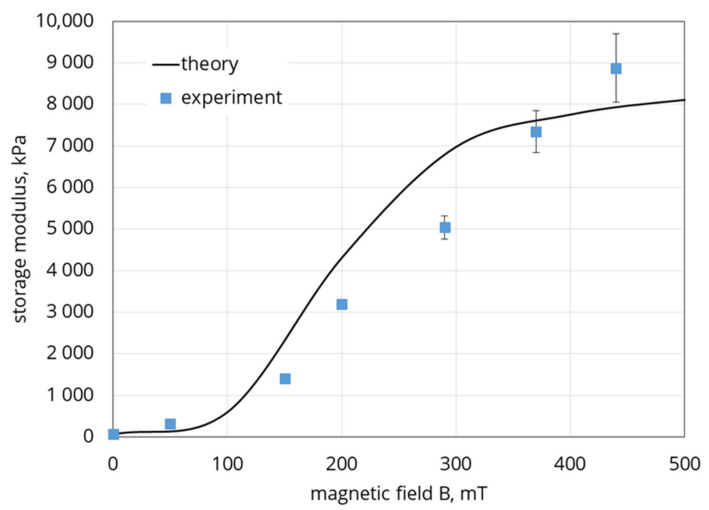
Shear modulus of the Fe/Ni alloy and carbonyl iron-based samples as a function of magnetic field (at the strain of 0.00014).

The dependence of the shear modulus on magnetic field strength has been studied. An MAE sample filled with the Fe75-Ni25 alloy was tested by the shear oscillation method using a rheometer HAAKE MARS III (Thermo Fisher Scientific, Waltham, MA, USA) equipped with a plate-plate measuring geometry (diameter of the rotating plate is 15 mm) and a custom-made magnetic cell. In order to secure the system from sample slipping between the planes of the measuring geometries, the elastomer was glued to the rotor and stator using a cyanoacryl glue. An axial (normal) force in the beginning of the measurement was set to 0 N in order to prevent any overlapping of the normal and shear stresses. In all tests, a constant frequency of oscillation of 1Hz was used. In Figure 6, an example of the dependence diagram of the measured shear modulus on the deformation magnitude (amplitude sweep) at various magnetic fields is presented. Figure 7 shows the magnetic field dependence of the averaged modulus obtained within independent measurements (at the strain of 0.00014). For comparison, the results obtained based on the carbonyl iron (30 vol. %) reference sample are additionally provided. 

The obtained results suggest that the shear modulus significantly depends on the value of shear deformation. The modulus grows as deformation weakens; the imposition of magnetic field only intensifies this tendency, which happens owing to the field-induced structuring of the filler particles inside the composite [41]. This behaviour is typical of filled elastic magnetic composites and corresponds qualitatively to all known results. On the other hand, in addition to the response to deformation, the strong field-dependence of the elastic properties of MAE also follows from the experimental data. For instance, the application of small deformations to the sample in a 440 mT magnetic field results in an increase of the elasticity modulus to ~8 MPa from the initial value of ~40 kPa, which means that the decrement amounted almost to 8 MPa or a factor of 200. Comparison of these results with those obtained for the carbonyl iron-based reference sample (Figure 7) as well as reported in the literature suggests that we observed one of the maximum effects ever reached in scientific investigations. It can be compared with the recently reported results [16], where at γ = 0.01%, G′ = 3–5 MPa; at γ = 0.1% G′ = 1–3 MPa; and at γ = 1%, G′ = 1–2.5 MPa in the 1T field. The reported MR effect was at γ = 0.01–58%, at γ = 0.1–300% and at γ = 1–409% [16].

**Figure 8:**
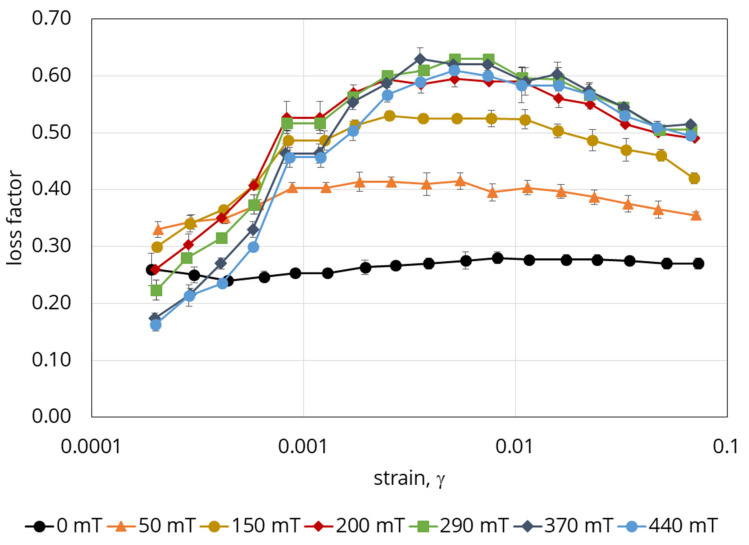
Dependence of the loss factor of the material on deformation (strain) and magnetic field for the Fe–Ni alloy-based sample.

A further result note is the dependence of the loss factor on the deformation magnitude and magnetic field strength (Figure 8).

**Figure 9:**
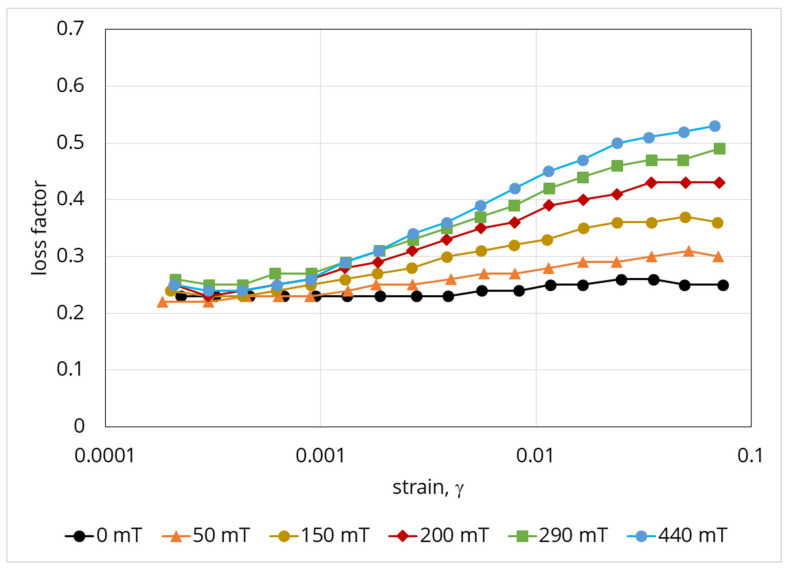
Dependence of the loss factor of the material on deformation (strain) and magnetic field for the reference carbonyl iron-based sample.

This diagram has two distinguished areas. The first one is the area of low deformations, at which magnetic field strengthening leads rather to the loss factor decreasing, while an increase in deformation leads to an increase in the loss factor. The transition area corresponds to the reaching of maximum loss factor values. The second area is the area of higher deformations, the main feature of which is that the loss factor decreases. The areas’ limits depend on the magnitude of the magnetic field applied for fields below 200 mT. This information suggests that in the designing of damping devices, the specific features of the behaviour of a given material must obviously be taken into account. If the object of interest is the area of high deformations, stronger fields are required for the appropriate damping of the system. If, however, variation of elasticity in magnetic field is the point of interest, the area of low deformations should be selected. Observed material behavior in respect to the loss factor, is related to the irregular shape of the magnetic fillers and, accordingly, significant internal friction in the particulate composite. To confirm this, Figure 9 shows the results obtained for a reference sample filled with carbonyl iron powder (Figure 1). The loss factor grows monotonically with increasing deformation, while in the area of small deformations, the applied field has almost no or minimal effect on it. By comparing the results presented in Figure 7, Figure 8 and Figure 9, one can conclude that the morphology of particles and mechanics of their interaction with the matrix in the external magnetic field has a significant role in the behaviour of the composite.

Our measurements considered here are limited to deformation not exceeding 10% and an important remark should also be made that rheometric measurements in the region of higher deformations will be clearly related to the region of nonlinear viscoelasticity. In this case, the module values indicated by standard commercial rheometer software will have no relation to the absolute physical properties of the material. 

Next, we propose a theoretical model of macroscopic shear deformation of soft magnetic elastomers polymerized without a magnetic field, that is, the corresponding experimentally studied samples.

## 4. Theoretical Model of the MR-Effect Internal Mechanism 

Experiments [53,54,55,56] demonstrate that in magnetic suspensions as well as in ferrogels, based on the solution of biological polymers, cured without an external magnetic field, particles of a magnetic filler form isotropic primary agglomerates and other heterogeneous structures. The appearance of these structures leads to strong dependence of the suspensions and ferrogels’ rheological properties on an applied magnetic field and volume concentration of the particles. The measured elastic and viscous modulus of these systems are significantly higher than the predictions of traditional models proposed for composite materials, which deal with individual particles, distributed in a host continuous medium. The concept of the primary agglomerates has recently allowed to explain quantitatively the observed strong concentration effect in the ferrogels out the field as well as MR effects in the liquid alginate suspensions [53,54]. The aim of this part of the current study is to propose a theoretical model explaining the observed experimentally very high MR effect (Figure 8). 

Following [53,54], we consider the MAE as composites, consisting of the elastic continuous medium and spherically-shaped primary agglomerates, composed of the individual particles of the magnetic filler.

### 4.1. Chain Formation

**Figure 10:**
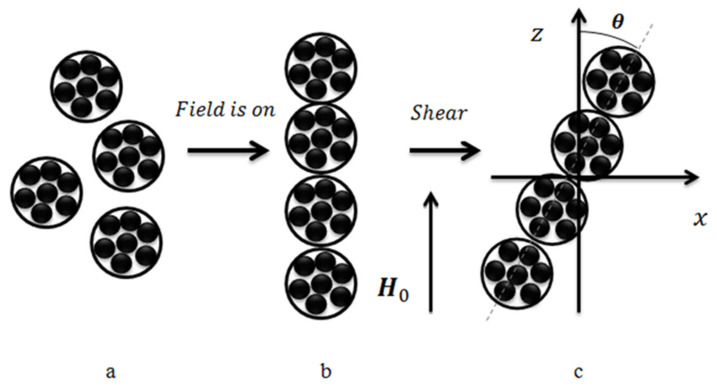
Sketch of the model system: (**a**) isotropic distributed spherical-shaped agglomerates; (**b**) agglomerates united into a linear chain aligned along an externally applied field; (**c**) deviation of the chain from the field direction under macroscopic shear deformation.

A sketch of the model system is shown in Figure 10. It is taken into account that the studied composites have been cured without an applied field. During polymerization, the magnetic particles formed the isotropic spherical-shaped agglomerates (Figure 10a). Then, after curing under the applied field, these agglomerates unite into linear chains aligned along an externally applied field (Figure 10b). Under the macroscopic shear deformation, the chains deviate from the field direction (Figure 10c).

Our first aim is to model the transformation from the state a to b in Figure 10 and to determine the average agglomerates number in the chains under the given magnetic field. For maximal simplification of the mathematical part of the problem, like in [53,54], we suppose that the primary agglomerates are identical spheres (see Figure 10a). 

Following [57,58], we will use the main ideas of the hierarchical model of the chain formation in magnetorheological materials (elastomers, gels and fluids), combined with the lattice model of the particles (agglomerates) disposition. Note that the lattice approach is often used in the statistical physics of gas and liquid systems [59].

**Figure 11:**
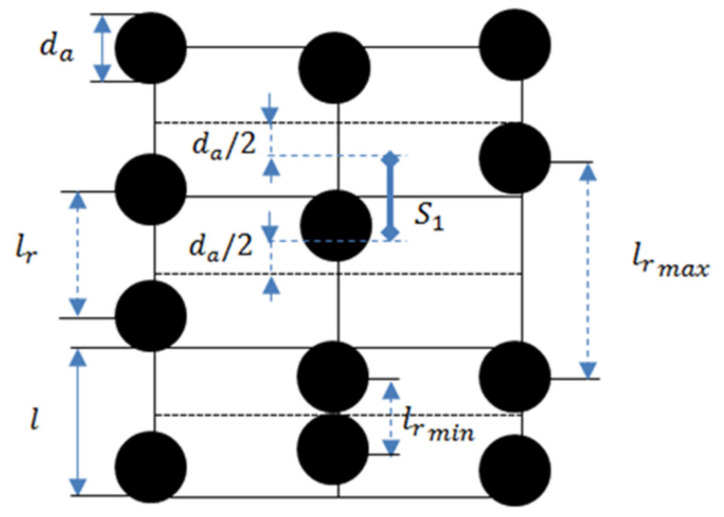
Sketch of the cubic lattice. The centers of each agglomerate can be located at any point within its segment *S_1_*.

Let us consider a cubic lattice, illustrated in Figure 11, with the edge length *l*. This length we estimate from the condition that the ratio of the agglomerate volume to the volume of the cell is equal to the agglomerates volume concentration Φ in the composite:

(1) $$l=d_{a}\cdot {\left(\frac{\pi }{6\Phi }\right)}^{\frac{1}{3}}$$

here, *d_a_* is the agglomerate diameter.

We will suppose that, before the field is applied, all agglomerate centers in the system are located at any point within a liner segment *S_1_ = l − d_a_* of a cell of the cubic lattice with an equal probability; the segment center coincides with the center of the lattice cell (Figure 11). We will denote the random distance between centers of the neighboring agglomerates as *l_r_*. The distance lies in a segment (see Figure 11):

(2) $$d_{a}\leq l_{r}\leq 2S_{1}+d_{a}$$

**Figure 12:**
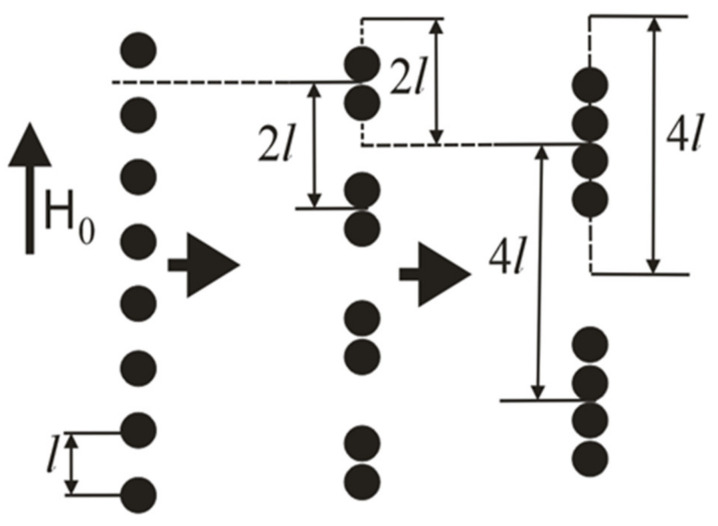
Sketch of three first stages (*k* = 0, 1, 2) of the agglomerates aggregation. The horizontal arrows illustrate evolution of the agglomerates in time. The segments of possible positions of the chains are shown. The segment’s boundaries are for the poles of the agglomerates at the chains’ extremities. The single agglomerates and the chains are shown in the centers of the segments of their possible positions.

Let us consider now the agglomerates unification into the chains, illustrated in Figure 12. In the hierarchical approach, we consider the chain formation as the unification of the single agglomerates into the doublets; then, unification of the doublets into the quartets of the agglomerates etc. Each chain consists of *n* = *2^k^* agglomerates, where *k* = 0.1 is the number of the stage of the chaining. Various stages of this unification are illustrated in Figure 12.

The number of agglomerates in the stable chain is determined by the competition between the forces of magnetic attraction between the agglomerates and the forces of the gel elastic resistance to the displacement of the agglomerates. For simplification of calculations, like in [57], we do not take into account the interaction of the agglomerates located on different axes of the lattice.

**Figure 13:**
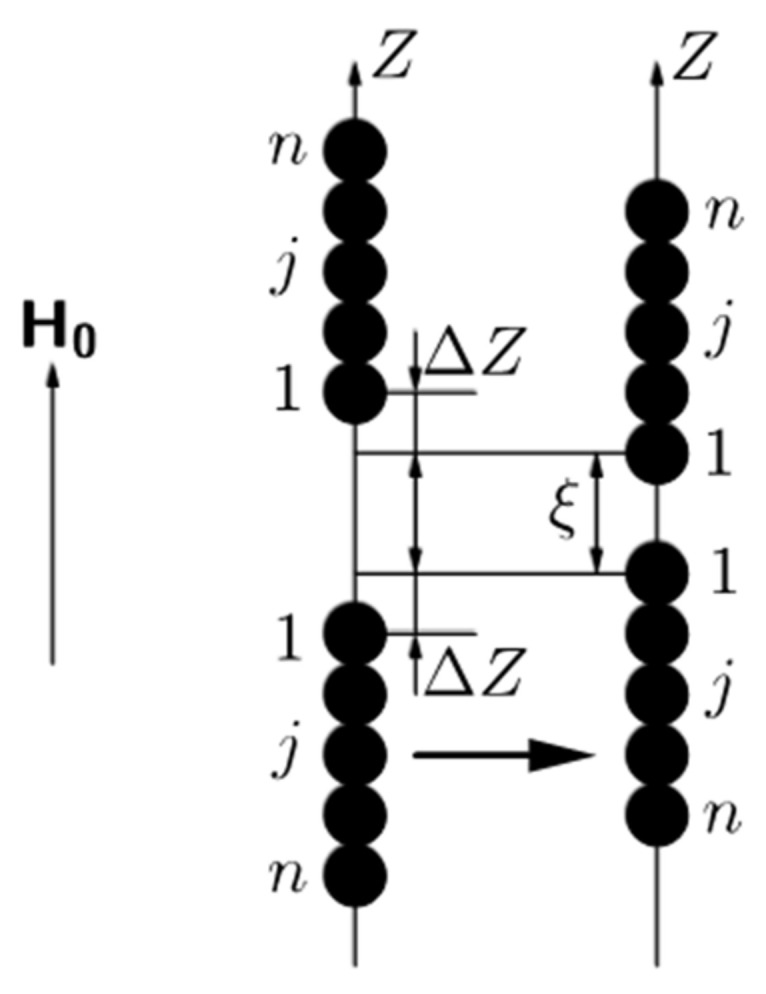
Illustration of the chains’ displacement towards each other. Left—the relative position of the chains immediately after their formation; right—after the displacement. Horizontal arrow—evolution in time.

We consider an arbitrary lattice axis, parallel to the magnetic field *H*_0_ (see Figure 12). At the beginning, the agglomerates are in single state, as it is illustrated in the left axis of Figure 12. Each single agglomerate’s center can be moved separately at any point within their own segment *S*_1_ then the center of the doublet, consisting of these two agglomerates, can be located at any point of a segment *S*_2_, the length of which is calculated as a sum of segments of the single agglomerates *S*_2_ = 2*S*_1_. A similar assumption is made for the centers of the four-agglomerate clusters, as shown in Figure 12. This unification algorithm can be continued further till the *n*-agglomerate chain (see Figure 13). The segment for the *n*-agglomerate chain equals to the sum of the n single agglomerate’s segments:

(3) $$S_{n}=n\cdot S_{1}=n\cdot \left(l-d_{a}\right)$$

Let us consider two neighbor *n*-agglomerate chains and denote a number of an agglomerate in the chain as *j*. We suppose that the “lowest” agglomerate in the “upper” *n*-agglomerate chain and the “highest” agglomerate in the “down” *n*-agglomerate chain (see illustration in Figure 13) have the number 1. Simple calculations show that displacement of the *j*-th agglomerate right after the *n*-agglomerate chain formation in the upper chain is:

(4) $$\delta z_{j}^{\left(0\right)}=\left(\frac{n+1}{2}-j\right)\left(l_{r}-d_{a}\right)$$

Let us suppose now that each of these two chains has been displaced (under their magnetic attraction) towards the other one for the distance ΔZ with respect to the position of the chain formation. This situation is illustrated in Figure 13.

Total displacement of the *j*-th agglomerates of the upper chain in Figure 13, with respect to the agglomerate initial position shown in Figure 11, is:

(5) $$\delta z_{j}=\delta z_{j}^{\left(0\right)}-\Delta Z=\left(\frac{n+1}{2}-j\right)\left(l_{r}-d_{a}\right)-\Delta Z.$$

Similarly, the total displacement of the *j*-th agglomerate in the down chain is:

(6) $$\delta z_{j}=-\delta z_{j}^{\left(0\right)}+\Delta Z=-\left(\frac{n+1}{2}-j\right)\left(l_{r}-d_{a}\right)+\Delta Z.$$

This is convenient to introduce the distance *ξ* between the centers of the agglomerates at the nearest extremities of the chains (see Figure 13). One can easily show that the following relation is held:

(7) $$\xi =L_{n}-2\Delta Z\;L_{n}=n\left(l_{r}-d_{a}\right)+d_{a},$$

here *L_n_* is the distance between centers of nearest agglomerates of the stable neighboring *n*-agglomerate chains; this distance directly depends on the initial (for single agglomerates) random distances *l_r_*. Using this formula and Equations (2) and (3), we obtain the range of values for this distance

(8) $$d_{a}\leq L_{n}\leq 2\cdot S_{n}+d_{a}$$

The total energy of the matrix deformation, corresponding to the positions of the chains shown in Figure 13, in the Hook approximation, can be presented as:

(9) $$U_{n}^{\left(el\right)}=\beta \overset{n}{\underset{j=1}{\sum }}\left[{\left(\delta z_{j}^{\left(0\right)}-\Delta Z\right)}^{2}+{\left(\Delta Z-\delta z_{j}^{\left(0\right)}\right)}^{2}\right],$$

here *β* = 3*πG*_0_*d_a_* and *G*_0_ is the shear modulus of the matrix [57].

Combining Equations (5)–(7) and (9), we come to the following estimate for the dimensionless elastic energy:

(10) $$U_{n}^{el}\left(\xi \right)=\frac{\beta }{2}\left[\overset{n}{\underset{j=1}{\sum }}{\left(\left(n+1-2j\right)\left(l_{r}-d_{a}\right)+L_{n}-\xi \right)}^{2}\right]$$

The elastic force, which resists the chains approaching, can be written as

(11) $$F_{n}^{el}\left(\xi \right)=-\frac{dU_{n}^{el}\left(\xi \right)}{d\xi }=\frac{\beta n}{2}\left(L_{n}-\xi \right).$$

### 4.2. Determination of the Mean Agglomerate Number in the Chains

Now we will estimate the magnetic force of interaction between the *n*-agglomerate chains (see Figure 11). In the framework of the simplest dipole-dipole approximation, this force can be presented as [57]:

(12) $$F_{n}^{m}\left(\xi \right)=-\frac{3{µ}_{0}}{2\pi }\overset{n}{\underset{i=1}{\sum }}\overset{n}{\underset{k=1}{\sum }}\frac{m_{i}m_{k}}{r_{ik}{}^{4}}$$

**Figure 14:**
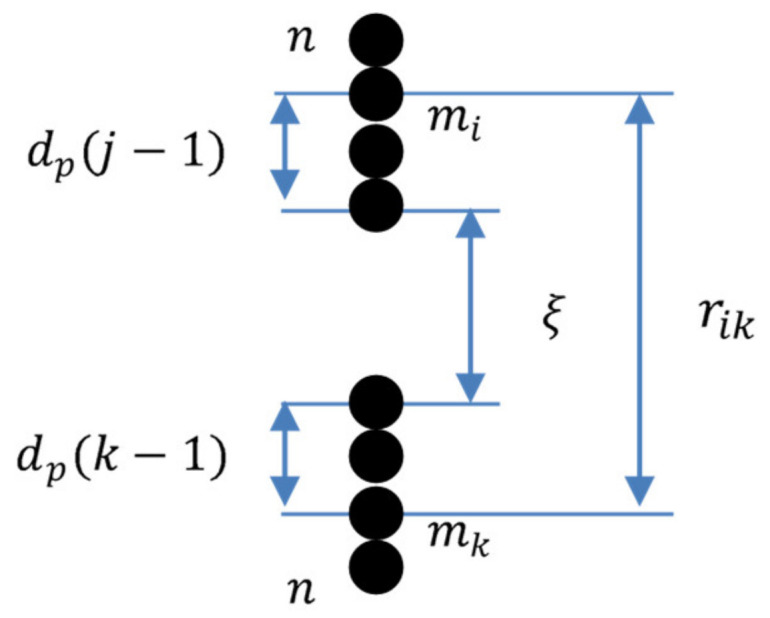
Illustration of the distance between centers of *k*-th and *j*-th agglomerates located in neighboring two *n*-agglomerate chains.

Here, *µ*_0_ is the vacuum magnetic permeability; *m_i_* is the magnetic moment of the *i*-th agglomerate in the chain; and *r_ik_* is the distance between centers of the *k*- and *j*-agglomerates (see Figure 14).

We will suppose that magnetic moments of all agglomerates in the chains are identical. The results of [57] show that this approximation leads to a slight deviation from a more accurate, but much more cumbersome approach, which accounts for dependence of the agglomerate magnetic moments on its position in the chain. In Equation (12), we will denote the moment of the agglomerate in the *n*-agglomerate chain as *m_n_ = V_a_M_n_*, where *V_a_* is the agglomerate volume and *M_n_* its magnetization. Substituting this relation into (12), taking into account *r_ik_* = *ξ* + *d_a_*(*i* + *k* − 2) (see Figure 14), we come to

(13) $$F_{n}^{m}\left(\xi \right)=-\frac{3{µ}_{0}V_{a}{}^{2}M_{n}{}^{2}}{2\pi }\overset{n}{\underset{i=1}{\sum }}\overset{n}{\underset{k=1}{\sum }}\frac{1}{{\left(\xi +d_{a}\left(i+k-2\right)\right)}^{4}}$$

**Figure 15:**
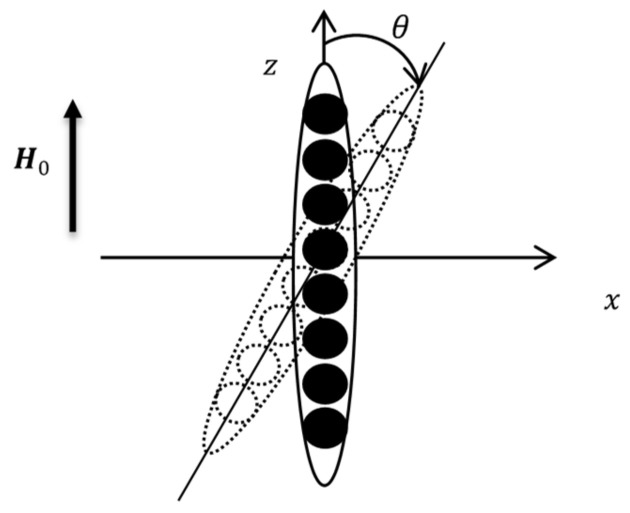
Illustration of modeling the chain-like aggregates as ellipsoids of revolution along the axis z. The chain parallel to the applied field corresponds to the non-deformed sample, and the declined chain to the sheared sample.

Because of the complicated shape of the chain, mathematically strict calculation of the magnetization *M_n_* is impossible. Here, to get physically transparent results, we will estimate *M_n_* for the *n*-agglomerate chain as magnetization of the ellipsoid of revolution with the major and minor axes *nd_a_* and *d_a_* respectively. This model of the chain in the non-deformed and sheared sample is illustrated in Figure 15. Note that this approach has been recently successfully used for calculations of rheological properties of liquid alginate suspensions with magnetic particles [53].

In the frame of the ellipsoidal model, magnetic field *H_in_* inside the chains can be determined using the classical relation of the magnetostatic theory [60]:

(14) $$H_{in∥}=H_{0}\cos\theta \;-M_{∥}N_{∥},\;H_{in⊥}=-H_{0}\sin\theta \;-M_{⊥}N_{⊥}.$$

Here, *θ* is angle deviation of the ellipsoid axis from the axis *z* due to the macroscopic shear deformation. The symbols ∥ and ⊥ mark the components of vectors parallel and perpendicular to the main axis of the ellipsoid; *N*_∥_ and *N*_⊥_ are the demagnetizing factors of the ellipsoid along and perpendicular to the axis [60]:

(15) $$N_{∥}=\frac{1}{n^{2}-1}\left[\frac{n}{\sqrt{n^{2}-1}}ln\left(n+\sqrt{n^{2}-1}\right)-1\right],\;N_{⊥}=\left(1-N_{∥}\right)/2.$$

Generally speaking, the agglomerate magnetization nonlinearly depends on the field *H*_0_. By using the semi-empirical Frohlich–Kennelly relation [61], we have:

(16) $$M=\chi \cdot H_{in},\;\;\chi =\frac{\chi_{0}\;M{\;}_{s}}{M{\;}_{s}+\chi_{0}H_{in}},$$

where $\chi_{0}$ and $M{\;}_{s}$ are initial susceptibility of the particle material and its saturated magnetization respectively; χ is the particle susceptibility in the internal field $H_{in}$.

In this section, we consider the non-sheared sample and put angle *θ* = 0 (see Figure 15). Combining Equations (14) and (16), one obtains

(17) $$M_{n}=M_{z}=\frac{\chi_{0}\;H{\;}_{0}+M_{s}\left(1+\chi_{0}N_{∥}\right)-\sqrt{{\left(\chi_{0}\;H_{0}+M{\;}_{s}\left(1+\chi_{0}N_{∥}\right)\right)}^{2}-4\chi_{0}{}^{2}M_{s}H_{0}N_{∥}}}{2N_{∥}\chi_{0}}$$

Let us write down the total force acting on the *n*-agglomerate chain as

(18) $$F_{n}\left(\xi ,L_{n}\right)=F_{n}^{m}\left(\xi \right)+F_{n}^{el}\left(\xi ,L_{n}\right)$$

The formation of a stable 2*n*-agglomerate chain from two *n*-agglomerate ones depends on the competition between the magnetic and elastic forces. Indeed, the agglomerates form chains because of magnetic attraction, however, elastic forces resist it. In Equations (11) and (12), these forces are negative and positive respectively. To create a new chain, the total force in (18) must be negative for all values *ξ*, *L_n_*.

Let us introduce the critical values $\xi_{cr}$, $L_{c}{}_{n}$ of these distances, which correspond to the aggregation of the *2n*-agglomerate chains under a given magnetic field (see illustration in Figure 14). These magnitudes can be found from the following conditions for the total force (see detailed explanation in [57]):

(19) $$F_{n}\left(\xi ,L_{n}\right)=0,\frac{dF_{n}\left(\xi ,L_{n}\right)}{d\xi }=0.$$

We denote the number of *n*-agglomerate chains in a unite volume of the system as *g_n_*. This distribution function satisfies the following normalization condition

(20) $$\overset{\infty }{\underset{i=1}{\sum }}ng_{n}=\frac{\Phi }{V_{a}}.$$

Note that the ratio $\frac{\Phi }{V_{a}}$ is a total number of the agglomerates in the composite unite volume.

The distance *L*_1_ *= l_r_* between the single agglomerates (before their aggregation) is probabilistic and the distance *L_n_* between the stable *n*-agglomerate chains depends on *l_r_* (see Equation (7)). With an equal probability, *L_n_* can have any value within the range of inequality (8). The agglomerates will unite into the chains when the inequality $L_{n}<L_{cr}{}_{n}$ is held. Taking it into account, we convert the inequality (8) as

(21) $$0\leq \frac{L_{cr}{}_{n}-d_{a}}{2\cdot S_{n}}\leq 1,\;\;P_{2n}=\frac{L_{cr}{}_{n}-d_{a}}{2\cdot S_{n}}.$$

The value $\frac{L_{cr}{}_{n}-d_{a}}{2\cdot S_{n}}$ is a probability *P*_2*n*_ of the 2*n*-agglomerate chain formation. The distribution function *g_n_* can be determined through the probability *P*_2*n*_ in the following way. Consider that the *n*-agglomerate chain is formed by unification of two *n/2*-agglomerate chains. In their turn, the *n/2*-agglomerate chain is formed as a result of unification of two *n/4*-agglomerate chains, etc. up to the single agglomerates (see Figure 12). Thus, to determine *g_n_*, all probabilities $P_{n},P_{n/2},\;P_{n/4},\ldots ,\;P_{2},\;P_{1}$ must be taken into account, and

$$g_{n}\;=\frac{\varphi }{nV_{p}}\left(1-P_{2n}\right)P_{n}P_{n/2}P_{n/4}\cdot \ldots \cdot P_{2}P_{1}=\frac{\varphi }{nV_{p}}\left(1-P_{2n}\right)\overset{n}{\underset{i=1}{\prod }}P_{i},$$

(22) $$g_{1}=\frac{\Phi }{V_{a}}\left(1-P_{2}\right)\;.$$

Here, the factor $\frac{\Phi }{V_{a}n}$ is the probability that only *n*-agglomerate chains take place in the system. Mathematically, this means that the multiplication of all probabilities till *i* = *n* equals to one (i.e., $P_{n}P_{n/2}P_{n/4}\cdot \ldots \cdot P_{2}P_{1}=1$) and *P*_2*n*_ = 0 when defining the number of *n*-agglomerate chains in a unite volume of the system; it is necessary to take into account the fact that a 2*n*-agglomerate chain must not be formed, therefore, using the definition of inverse probability from probability theory we write in this formula the factor (1 − *P*_2*n*_) that means the inverse probability that the 2*n*-agglomerate chains are formed.

Remember that *g_n_* is the number of the *n*-agglomerate chain in the unite volume of the system. By using (20), we can determine the mean agglomerate number *<n>* in the chains:

(23) $$<n>=\frac{\sum_{i=1}^{\infty }ng_{n}}{\sum_{i=1}^{\infty }g_{n}}=\frac{\varphi }{V_{a}}\frac{1}{\sum_{i=1}^{\infty }g_{n}}$$

**Figure 16:**
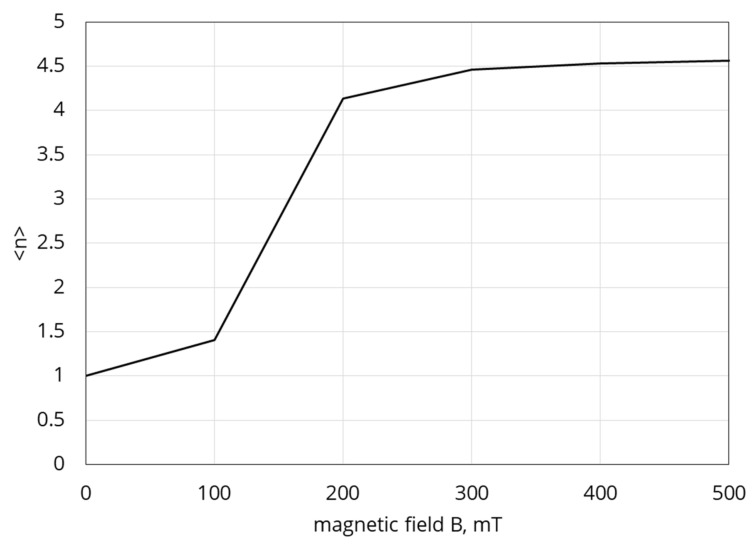
The mean number $<n>$ of agglomerates in the chains vs. applied magnetic field induction *B*. System parameters: $\chi_{0}=300,\;G_{0}=10\;\mathrm{kPa},\;M_{s}=820\;k\frac{A}{m},\;d_{a}=20\;µm,\Phi =0.3$.

We have solved system (19) numerically and, combining (22) and (23), determined the mean number *<n>* of agglomerates per chain. Some results of our calculations are presented in Figure 16.

### 4.3. The Shear Modulus

We consider the MRE specimen as a composite consisting of the elastic matrix and identical elongated magnetizable chains; the number of primary agglomerates in the chain is the mean number <*n*>, defined in Equation (23). Let the composite experience a small macroscopic shear perpendicular to the field (as it is shown in Figure 10c. Following [54] and the general theory of mechanics of polar media (see, for example, [60,62,63,64]), the shear modulus of the composite is when presented as:

(24) $$G=G_{s}+G_{a}$$

Here, the first term corresponds to the symmetrical stress, which appears due to elastic deformations in the host matrix; the second term corresponds to the anti-symmetrical stress, induced by magnetic torques, acting on the chains. To estimate *G_s_*, we have used the Krieger–Dougherty relation [65], successfully used before for calculations of the viscosity and viscoelastic moduli of liquid alginate magnetic suspensions and cured ferrogels [53,54,66]:

(25) $$G_{s}\left(n\right)=G_{0}{\left(1-\frac{\Phi }{\Phi_{m}}\right)}^{-\left[G\left(n\right)\right]\Phi_{m}},$$

where Φ*_m_* is the volume fraction of the dense packing; [*G(n)*] is determined, like in [53], from the condition that the complex *G*_0_(1 + [*G(n)*]Φ) is equal to the modulus of extremely diluted (Φ << 1) composite.

To estimate [*G(n)*], we model, similar to [53], the chain-like aggregates as ellipsoids of revolution, illustrated in Figure 15, with the minor and major axes equal to *d_a_* and *nd_a_*, respectivly. As a result, we come to the relation (see details in [53]):

(26) $$\left[G\left(n\right)\right]=\alpha \left(n\right)+\frac{\zeta \left(n\right)+\beta \left(n\right)\lambda \left(n\right)+\beta \left(n\right)}{2}+\chi \left(n\right)-2\beta \left(n\right)\lambda \left(n\right)$$

Here, *α(n)*, … *λ(n)* are shape-factors of the ellipsoid, given in Appendix A. It was shown in [67] that the effect of the individual chains on macroscopic properties of the composites dominates over the effects of inter-chain interaction. That allows us to apply Equation (26) for all agglomerate volume fractions presenting interest.

Since the agglomerates in a chain are composed of magnetic particles, we must estimate the volume fraction Φ of these aggregates. This volume concentration cannot be calculated theoretically, because it is determined by the uncontrolled details of the composite synthesis. However, it can be estimated from the experiments without applied field, when the agglomerates can be considered as individual spheres, shown in Figure 10a. By using Equation (25), one gets:

(27) $$\Phi =\left(1-{\left(\frac{G_{s}{}_{exp}\left(H_{0}=0\right)}{G_{0}}\right)}^{\frac{-1}{\left[G\left(1\right)\right]\Phi_{m}}}\right)\Phi_{m}.$$

Here, [*G*(1)] = 5/2 (see Equation (26)) is the classical Einstein multiplier, related to the system of single spheres. Experiments provide $G_{s}{}_{exp}\left(H_{0}=0\right)=6.3\;\mathrm{kPa}$. This gives Φ ≈ 0.437.

Since only small shear deformations are considered here, it is reasonable to suppose that the angle *θ*, illustrated in Figure 15, of the chain axis deviation from the field *H*_0_ is also small. Let us consider the ellipsoid deviated from the direction of the applied magnetic field by the very small angle *θ* (Figure 15). By using $H_{in∥}\equiv H_{in}$ in Equation (14), one can estimate the anti-symmetrical part *G_a_* in Equation (24) as (see also [53]):

(28) $$G_{a}=\frac{{µ}_{0}\Phi H_{0}{}^{2}}{2}\frac{M_{n}{}^{2}\left(N_{∥}-N_{⊥}\right)}{(H_{in}+M_{n}N_{∥})\left((H_{in}+M_{n}N_{⊥}\right)}$$

**Figure 17:**
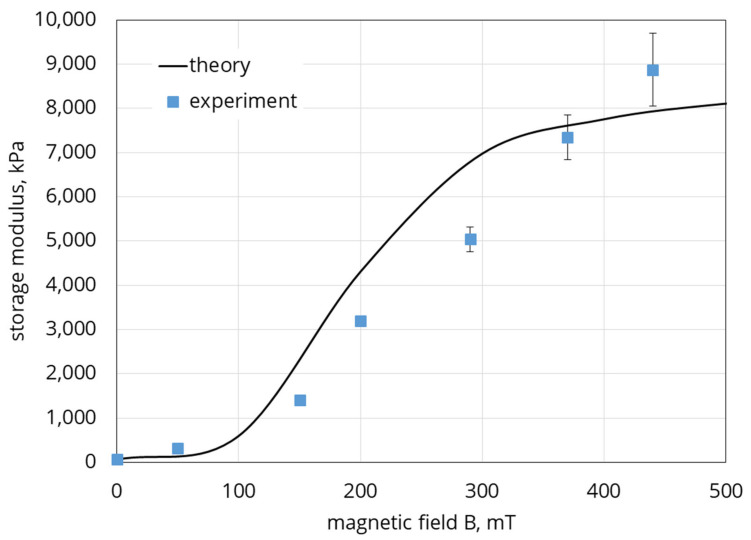
Shear modulus vs. the applied magnetic field. System parameters: $\chi_{0}=300,\;G_{0}=10\;\mathrm{kPa},\;M_{s}=820\;k\frac{A}{m},\;d_{a}=20\;µm,\Phi =0.437,\Phi_{m}=0.63$. Line—theory, squares—experimental data shown in Figure 7. Volume concentration of the embedded particles is 30%.

Combining Equations (23), (26)–(28), we calculate the shear modulus of the composite in Equation (28). A comparison of the calculations with the experimental date from Figure 8 is shown in Figure 17.

The reasonable agreement between theoretical and experimental results demonstrates that the proposed model is adequate, at least, in its main physical points. It should be noted that the model considers the quasi-static deformation of the sample, while in the experiment the behavior of the material was investigated in the oscillatory test. Nevertheless, the experiment used an extremely low oscillation frequency and the comparison seems to be quite acceptable. Obviously, further development of the model capable of predicting the dynamic response of the composite is required.

## 5. Conclusions and Outlook

The obtained mechanochemical permalloy shows high magnetization, consists of particles of elliptical or irregular shape, and is highly effective as a filler for MAEs/MREs. Efficiency is determined by a higher average particle size of the order of 20 µm, which is a high polydispersity (or binary size distribution) of an irregular elliptical powder shape. The elastomer has a high loss modulus due to irregular shape of the magnetic fillers and, accordingly, significant internal friction. A high relative change in the elastic modulus of about 200 times was obtained, while its absolute increase can be up to ~8 MPa. This makes MAE based on the permalloy powder very attractive for damping applications. On the other hand, the morphology and particle size of the powder may be a disadvantage in terms of durability of the composite, since irreversible changes in the microstructure of the polymer are possible due to movement and rotation of the particles inside the matrix. This issue requires special study. Also, in further works, the question of frequency dependencies of material behavior should be addressed. Attention should also be paid to the possible influence of the effect of magneto-deformation (striction) of the magnetic elastic composite on the results of measuring viscoelastic properties. Stresses arising in the material due to this effect can interact with shear stress when the material is under oscillating strain. 

Furthermore, a physical model is proposed that allows to predict the MR effect in the considered composite adequately enough. The model is based on the effect of particle chains formation inside the magnetic elastomer. It is not the chains of individual particles that are considered, but chains consisting of larger aggregates. Taking into account a real morphology of particle structures in magnetic polymeric materials, see e.g., [68,69], this model is more realistic in terms of applicability to highly filled composites. The effect is most pronounced in the region of small strains, since structures are partially destroyed at large strains. The results of the theoretical calculation give a quantitative agreement with the experiment, although the model considers the situation of quasi-static loading. To predict the dynamic response of the material, further development of the model is necessary. Note that the significant MR effects in ferrogels have also been detected in [57]. However, our experience shows that macroscopic rheological effect in these systems strongly depends on the condition of their synthesis, because these conditions determine morphology of internal heterogeneous structures in ferrogels. That is why theoretical analysis of the results obtained in other experimental works requires special detailed studies.

## Appendix A

The shape factors $\alpha \left(n\right),\ldots$ introduced in Equation (26), are [56]:

$$\alpha \left(n\right)=\frac{1}{n\alpha_{0}^{\prime }}$$

$$\beta \left(n\right)=\frac{2\left(n^{2}-1\right)}{n\left(n^{2}\alpha_{0}+\beta_{0}\right)}$$

$$\zeta \left(n\right)=\frac{4}{n\left(n^{2}+1\right)\beta_{0}^{\prime }}-\frac{2}{n\alpha_{0}^{\prime }}$$

$$\chi \left(n\right)=\frac{2\alpha_{0}^{\prime \prime }}{n\alpha_{0}^{\prime }\beta_{0}^{\prime \prime }}-\frac{8}{n\beta_{0}^{\prime }\left(n^{2}+1\right)}+\frac{2}{n\alpha_{0}^{\prime }}$$

$$\lambda \left(n\right)=\frac{n^{2}-1}{n^{2}+1}$$

where

$$\alpha_{0}=-\frac{1}{n^{2}-1}\left[\frac{2}{n}+\frac{1}{\sqrt{n^{2}-1}}\ln\left(2n^{2}-1-2n\sqrt{n^{2}-1}\right)\right]$$

$$\beta_{0}=\frac{1}{n^{2}-1}\left[n-\frac{1}{2\sqrt{n^{2}-1}}\ln\left(2n^{2}-1+2n\sqrt{n^{2}-1}\right)\right]$$

$$\alpha_{0}^{\prime }=\frac{1}{4{\left(n^{2}-1\right)}^{2}}\left[n\left(2rn^{2}-5\right)-\frac{3}{2\sqrt{n^{2}-1}}\ln\left(2n^{2}-1-2n\sqrt{n^{2}-1}\right)\right]$$

$$\beta_{0}^{\prime }=\frac{1}{{\left(n^{2}-1\right)}^{2}}\left[\frac{n^{2}+2}{n}-\frac{3}{2\sqrt{n^{2}-1}}\ln\left(2n^{2}-1+2n\sqrt{n^{2}-1}\right)\right]$$

$$\alpha_{0}^{\prime \prime }=\frac{1}{4{\left(n^{2}-1\right)}^{2}}\left[n\left(2n^{2}+1\right)-\frac{4n^{2}-1}{2\sqrt{n^{2}-1}}\ln\left(2n^{2}-1+2n\sqrt{n^{2}-1}\right)\right]$$

$$\beta_{0}^{\prime \prime }=-\frac{1}{{\left(n^{2}-1\right)}^{2}}\left[3n+\frac{2n^{2}+1}{2\sqrt{n^{2}-1}}\ln\left(2n^{2}-1-2n\sqrt{n^{2}-1}\right)\right]$$
