# Correction: Borin, D., et al. Magnetorheological Effect of Magnetoactive Elastomer with a Permalloy Filler. *Polymers* 2020, *12*, 2371

**DOI:** 10.3390/polym13020172

**Published:** 2021-01-06

**Authors:** Dmitry Borin, Gennady Stepanov, Anton Musikhin, Andrey Zubarev, Anton Bakhtiiarov, Pavel Storozhenko

**Affiliations:** 1 Chair of Magnetofluiddynamics, Measuring and Automation Technology, TU Dresden, 01069 Dresden, Germany; 2 State Scientific Research Institute for Chemical Technologies of Organoelement Compounds, Shosse Entuziastov 38, 111123 Moscow, Russia; gstepanov@mail.ru (G.S.); abakhtia@gmail.com (A.B.); bigpastor@mail.ru (P.S.); 3 Department of Theoretical and Mathematical Physics, Ural Federal University, Lenina Ave 51, 620083 Ekaterinburg, Russia; antoniusmagna@yandex.ru (A.M.); A.J.Zubarev@urfu.ru (A.Z.); 4 M.N. Mikheev Institute of Metal Physics of the Ural Branch of the Russian Academy of Sciences, 620108 Ekaterinburg, Russia

The authors wish to make a change to the published paper [1]. In the original manuscript, the authors made a mistake when proofreading and added an incorrect Figure 7. The corrected Figure 7 is presented below.

The authors apologize for any inconvenience caused and the change does not affect the scientific results. The manuscript will be updated, and the original will remain online on the article webpage at https://www.mdpi.com/2073-4360/12/10/2371.

## Figures and Tables

**Figure 7:**
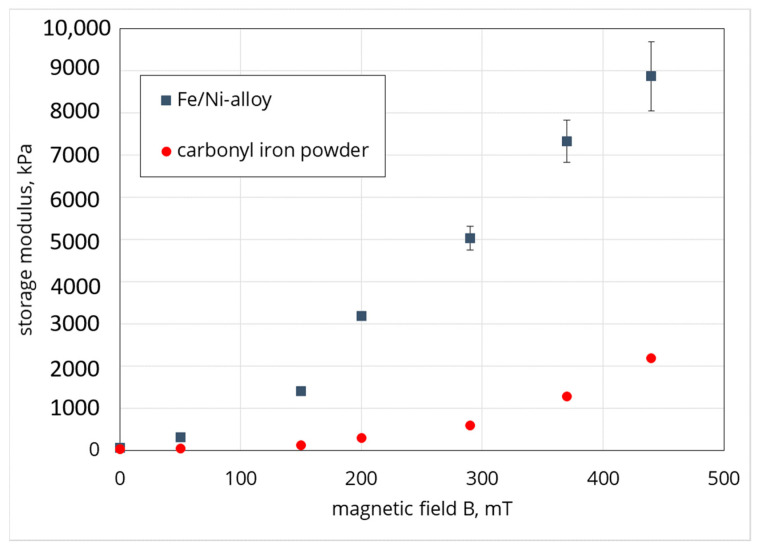
Shear storage modulus of the Fe/Ni alloy and carbonyl iron-based samples as a function of magnetic field (at a strain of 0.00014).
